# Increase of Th17 Cell Phenotype in Kidney Transplant Recipients with Chronic Allograft Dysfunction

**DOI:** 10.1371/journal.pone.0145258

**Published:** 2015-12-30

**Authors:** Byung Ha Chung, Kyoung Woon Kim, Bo-Mi Kim, Kyoung Chan Doh, Mi-La Cho, Chul Woo Yang

**Affiliations:** 1 Convergent Research Consortium for Immunologic disease, St. Mary's Hospital, College of Medicine, The Catholic University of Korea Seoul, Seoul, Korea; 2 Transplant research center, St. Mary's Hospital, College of Medicine, The Catholic University of Korea Seoul, Seoul, Korea; 3 Division of Nephrology, Department of Internal Medicine, Seoul St. Mary's Hospital, College of Medicine, The Catholic University of Korea Seoul, Seoul, Korea; University of Toledo, UNITED STATES

## Abstract

This study was performed to determine the association of Th17 cell phenotype with chronic allograft dysfunction in kidney transplant recipients (KTRs). We compared the expression of Th17 cell phenotype in KTRs with chronic allograft dysfunction group (CAD, n = 52) with four control groups (long-term stable KTRs (LTS, n = 67), early stable KTRs (ES, n = 28), end stage renal disease (ESRD, n = 45), and healthy control (HC, n = 26). We also performed in vitro study using human proximal renal tubular epithelial cell line (HPRTEpiC) to evaluate the effect of IL-17 on human renal tubular epithelial cells. The CAD group showed increased percentage of Th17 cells out of CD4^+^ T cells and also increased proportion of IL-17 producing cells out of effector memory T cells or out of CCR4^+^CCR6^+^/CD4^+^ T cells compared to the LTS group and other control groups. Also, the serum level of IL-17, IL-33, and RAGE, and the expression of IL-1beta, RAGE, and HMGB1 mRNA showed an increase in the CAD group compared to the LTS group. In vitro study revealed that IL-17 increased production of IL-6 and IL-8 and up-regulated profibrotic gene expression such as ACTA-2 and CTGF in HPRTEpiC in a dose-dependent manner, which suggests that IL-17 has a role in the development of renal tubular cell injury. The results of our study may suggest that increase of Th17 cell phenotype could be a marker for the chronic allograft injury; hence there is a need to develop diagnostic and therapeutic tools targeting the Th17 cells pathway.

## Introduction

After kidney transplantation, CD4^+^ T cell mediated allo-immune responses play a crucial role in the development of chronic allograft rejection and dysfunction. Indeed, there is consistent evidence to support the involvement of specific populations of CD4^+^ T cells in the acceptance or rejection of the allograft by the host immune system [1,2,3,4,5,6]. Therefore, understanding the activation or suppression of a specific CD4^+^ T cell subset in kidney transplant recipients (KTRs) according to their clinical status, would be helpful to unveil the individual contributors to the progression of chronic allograft dysfunction.

Meanwhile, Th17 is the most recently discovered CD4^+^ T cell subset and it is characterized by the production of the pro-inflammatory cytokine IL-17 [7,8]. Accumulating evidences showed that Th17 cells are involved in driving immune processes previously thought to be exclusively Th1 mediated in various autoimmune diseases [9,10,11,12]. In addition, ongoing recent studies suggested that activation of Th17 cells may play a role in the development of allograft injury in organ transplantation [13,14,15,16,17]. Our previous studies also showed the clinical significance of increased Th17 infiltration in rejected allograft tissue or increased proportion of Th17 cells in the peripheral blood of KTRs [18,19,20].

In this regard, the aim of this study is to investigate the significance of the Th17 cell pathway in the progression of chronic allograft dysfunction in KTRs. Therefore, in this study, we evaluated the T cell immune profile including Th17 cells in patients with chronic allograft dysfunction compared to long-term allograft survivors with favorable allograft function and control groups such as stable KTRs with a short-term follow-up period, end stage renal disease (ESRD), and healthy controls (HC).

## Materials and Methods

### Patients and clinical information

**Fig 1:**
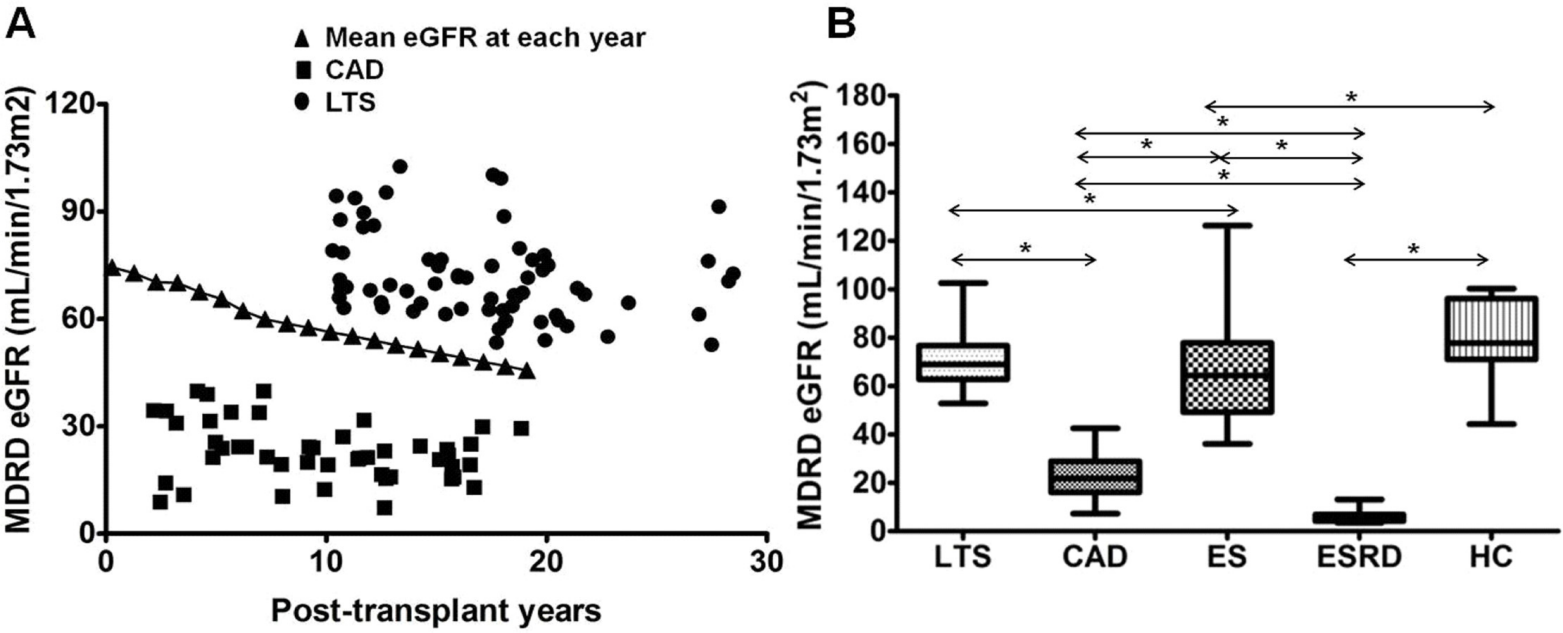
Distribution of allograft function in each study group. **(A)** Distribution of subjects according to allograft function and post-transplant years in LTS group and CAD group. Closed triangle mean the average value of MDRD eGFR at each post-transplant year in 587 patients who took kidney transplantation between 1995 and 2010 and current laboratory data is available in our center. **(B)** Comparison of allograft function assessed by MDRD eGFR in each study and control group. Note that allograft function was significantly superior in LTS group compared to CAD group. * *P*<0.05 for each comparison. LTS, long term stable; CAD, chronic allograft dysfunction; ES, early stable; ESRD, end stage renal disease; HC, healthy control; MDRD eGFR; Modification of Diet in Renal Disease estimated glomerular filtration rate.

Before defining each group, we investigated the yearly change in the average value of estimated glomerular filtration rate (eGFR) calculated by Modification of Diet in Renal Disease (MDRD) equation in 587 patients who underwent kidney transplantation between 1995 and 2010 and the current laboratory data is available at our center (Fig 1A). Based on the results, the definition of the long-term stable group (LTS group) was patients who were at least 10 years post-transplantation and showed higher MDRD eGFR than the mean value at each concordant post-transplant year. The definition of the chronic allograft dysfunction (CAD) group was KTRs who were at least 2 years post-transplantation and showed MDRD eGFR less than 40 mL/min/1.73m^2^ and histological evidence of IF/TA (TA [ct≥1] and IF [ci≥1] involving more than 25% of the cortical area) [21]. Another three control groups were included; KTRs with a follow-up duration of less than 6 months after KT and showed stable clinical course were included in the early stable (ES) control group; End-stage renal disease (ESRD) patients who were on hemodialysis or peritoneal dialysis for at least 3 months were included in the ESRD group, and healthy volunteers who showed normal renal function without underlying renal disease were included in the healthy control (HC) group. Table 1 shows the baseline clinical characteristics of included patient population and Fig 1B shows the distribution of MDRD eGFR in each group. This study was approved by the Institutional Review Board (KC10SISI0235) of the Seoul St. Mary’s Hospital, and written informed consent was obtained from all patients.

**Table 1:** Baseline characteristics of the patient populations.

	LTS (n = 67)	CAD (n = 52)	ES (n = 28)	ESRD (n = 45)	HC (n = 26)
**Age (year)**	55.2±9.7	48.3±8.6	41.2±10.3	44.7±10.5	35.5±9.3
**Male, n (%)**	38 (57)	25 (48)	16 (57)	29 (64)	8 (30.8)
**Post-transplant year**	17.1±4.9*,^†^	9.2±4.3^#^	0.4±01	-	-
**HLA mismatch**	3.1±1.4	3.3±1.5	3.1±1.9	-	-
**Donor (LD / DD), n (%)**	61 / 6 (91 / 9)*	31 / 21 (60 / 40)^†^	28 / 0 (100 / 0)	-	-
**Immune suppressant regimen**	*,^†^	^†^			
Tac based triple therapy	2 (3)	19 (38)	21 (75)	-	-
CsA based triple therapy	17 (25)	13 (26)	5 (18)	-	-
Tac based dual therapy	5 (8)	1 (2)	0 (0)	-	-
CsA based dual therapy	4 (6)	0 (0)	0 (0)	-	-
AZA+steroid	5 (8)	1 (2)	0 (0)	-	-
Tac monotherapy (±steroid)	3 (5)	9 (18)	2 (7)	-	-
CsA monotherapy (±steroid)	31 (46)	7 (14)	0 (0)	-	-

ADPKD, autosomal dominant polycystic kidney disease; AZA, azathioprine; CAD, chronic allograft dysfunction; CsA, cyclosporine; DD, deceased donor; DM, diabetes mellitus; ES, early stable; ESRD, end stage renal disease; GN, glomerulonephritis; HC, healthy control; LD, living donor; LTS, long term stable; MIZ, miroribine; MMF, mycophenolate mofetil; Tac, Tacrolimus; SRL, sirolimus

Tac (or CsA) based triple therapy included Tac (or CsA)+MMF+Steroid or Tac (or CsA)+MIZ+Steroid or Tac (or CsA)+SRL+Steroid

Tac (or CsA) based dual therapy included Tac (or CsA) +AZA, Tac (or CsA) +MMF, Tac (or CsA) +MIZ

* P<0.05 vs. CAD

^†^ P<0.05 vs. ES

^#^ P<0.05 vs. LTS

### Isolation and culture of immune cells from peripheral blood mononuclear cells

We collected peripheral blood for the analysis of immune cell profile and processed as follows. Peripheral blood mononuclear cells (PBMC) were prepared from heparinized blood by Ficoll–Hypaque (GE Healthcare, PA) density-gradient centrifugation. Cell cultures were performed as described previously [22]. In brief, the cell suspension was adjusted to a concentration of 10^6^/ml in RPMI1640 medium supplemented with 10% fetal calf serum, 100 U/mL penicillin, 100 mg/mL streptomycin, and 2 mM l-glutamine. The cell suspension (1 mL) was dispensed into 24-well multiwell plates (Nunc, Roskilde, Denmark).

### Flow cytometric analysis

Flow cytometric study of PBMC was performed within a few hours after sampling of the peripheral blood in a fresh state. For cytokine detection at the single-cell level, PBMCs were stimulated with 50 ng/mL phorbol myristate acetate (PMA) and 1 μg/mL ionomycin in the presence of GolgiStop (BD Biosciences, San Diego, CA) for 4 hours. For intracellular staining, cells were stained with combinations of the following mAbs: CD4–PE/Cy7 (RPA-T4, IgG1; BioLegend, San Diego, CA); CD8-APC (RPA-T8, IgG1,κ; Pharmingen, San Diego, CA); CD45RA–FITC (HI100, IgG2b, κ; Pharmingen, San Diego, CA); and CD25–APC (M-A251, IgG1, κ; Pharmingen). Staining for chemokine receptors was performed using the following mouse mAbs (all produced by Pharmingen): anti-CCR4 (1G1, IgG1), anti-CCR6 (11A9, IgG1), and anti-CCR7 (3D12, IgG2a, κ). Cells were washed, fixed, permeabilized and stained to detect intracellular cytokines with mAbs to IL-17 (PE, eBio64dec17, IgG1, κ; eBioscience, San Diego, CA); interferon (IFN)-γ (FITC, 4S.B3, IgG1, κ; eBioscience); IL-4 (APC, MP4-25D2, IgG1, κ; eBioscience); IL-17 (FITC (eBio64DEC17, IgG1, κ; eBioscience); Foxp3 (FITC, PCH101, IgG2a, κ; eBioscience); and IFN-γ (PE, B27, IgG1, κ; Pharmingen). Appropriate isotype controls were used for gate setting for cytokine expression. Cells were analyzed on a FACS Calibur flow cytometry system (BD Biosciences).

### Real-time reverse transcription polymerase chain reaction (RT-PCR)

mRNA was extracted from PBMCs using the TRIzol Reagent (Molecular Research Center, Inc, Cincinnati, OH), according to the manufacturer’s instructions. cDNA was synthesized in a PerkinElmer Cetus DNA thermal cycler (PerkinElmer, Inc, Waltham, MA) using the SuperScript Reverse Transcription system (Takara).

### Real-time PCR

A LightCycler 2.0 instrument (Roche Diagnostics; software version 4.0) was used for PCR amplification. All the PCR reactions were performed using LightCycler FastStart DNA Master SYBR Green I (Takara), according to the manufacturer’s instructions. The following primers were used for each molecule: for IL-1β, 5’- GCA CGA TGC ACC TGT ACG AT-3’(sense) and 5’- GGA GGT GGA GAG CTT TCA GTT C-3’(antisense); for RAGE, 5’- CCT CCC TGC AGA AAG CAC TT-3’(sense) and 5’- CCT CGG GTT CTG GGA AAA G-3’(antisense); for HMGB1, 5’- TGC TCT GAG TAT CGC CCA AA-3’(sense) and 5’- TCC TTC AGC TTC GCA GCC T-3’(antisense); for ACTA-2, 5’- GAG AAG AGT TAC GAG TTG CCT GAT G-3’(sense) and 5’- GTT GTA GGT GGT TTC ATG GAT GC-3’(antisense); for CTGF, 5’- GGA AAA GAT TCC CAC CCA AT-3’(sense) and 5’- TGC TCC TAA AGC CAC ACC TT-3’(antisense); and for β-actin, 5’-GGA CTT CGA GCA AGA GAT GG-3’(sense) and 5’-TGT GTT GGG GTA CAG GTC TTTG-3’(antisense). Housekeeping genes (β-actin) were amplified for normalization. Heat-map images were analyzed using the template designated by the manufacturer.

### Enzyme-linked immunosorbent assay (ELISA)

IL-17, IL-33, and RAGE levels in the serum from patients in the LTS and CAD groups were measured using sandwich ELISA (R&D Systems) according to the manufacturer’s instructions. IL-6 and IL-8 levels in the culture supernatants were measured using sandwich ELISA (R&D Systems) according to the manufacturer’s instructions. Absorbance at 405 nm was measured using an ELISA microplate reader (Molecular Devices).

### In vitro study using human renal proximal tubular epithelial cells

To evaluate the role IL-17 in acute and chronic renal tubular epithelial cell injury, we performed in vitro study. Human renal proximal tubular epithelial cells (HRPTEpiCs) were purchased from ScienCell Research Laboratories (ScienCell, CA, USA). HRPTEpiCs were maintained in epithelial cell medium (EpiCM, ScienCell Research Laboratories) supplemented with 2% fetal bovine serum (FBS), at 37°C with 5% CO_2_. HRPTEpiCs were seeded in 24 well plates at a density of 2x10^5^ HRPTEpiCs /ml in the above described medium. After 24 hours, the medium was replaced with HRPTEpiCs culture medium with or without addition of cytokines. HRPTEpiCs were stimulated with various doses of recombinant human IL-17 (rhIL-17, R&D Systems, Inc. Minneapolis, MN)(10, 50, 100 ng/ml) for 72 h. Supernatants were harvested and stored at –80°C until analysis.

### Statistical analysis

Statistical analysis was performed using SPSS software (version 16.0; SPSS Inc., Chicago, IL). The comparison of values among each group was made using one-way analysis of variance. For categorical variables, chi-square frequency analysis was used. The results are presented as mean ± standard deviation (SD). *P* values < 0.05 were considered significant.

## Results

### Comparison of IL-17 producing cells out of CD4^+^ T cells

**Fig 2:**
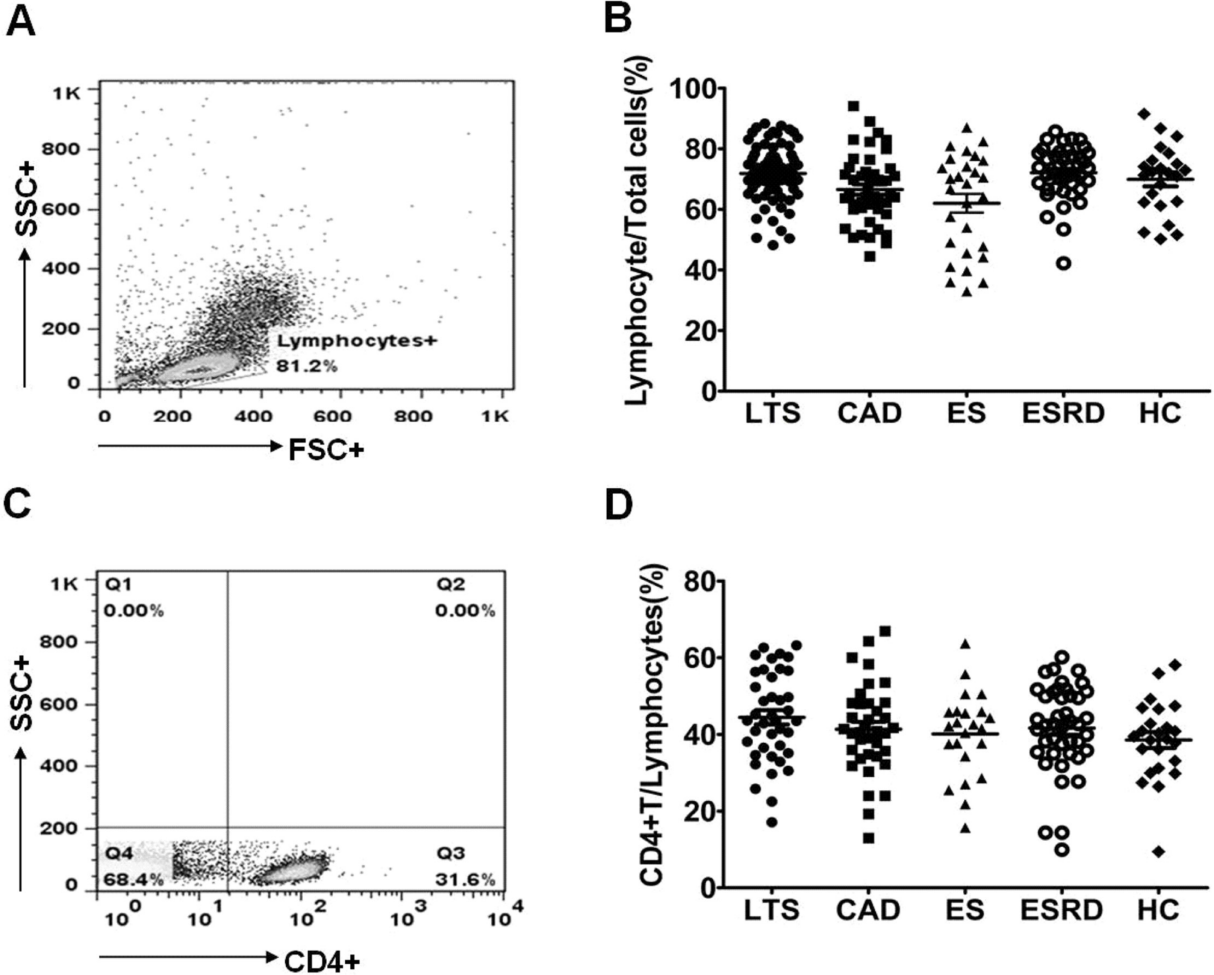
Distribution of lymphocyte and CD4^+^ T cell in each patient group. PBMC from each patient group were stimulated for 4h ex vivo with PMA and ionomycin in the presence of Golgi Stop. The percentage of target cells was measured by flowcytometry. **(A)** The representative figure of flowcytometric analysis for lymphocyte and CD4^+^ T cell. **(B)** The proportion (%) of Lymphocyte/Total cells and **(C)** CD4^+^ T/Lymphocyte cells in each group. * *P*<0.05 for each comparison. LTS, long term stable; CAD, chronic allograft dysfunction; ES, early stable; ESRD, end stage renal disease; HC, healthy control.

**Fig 3:**
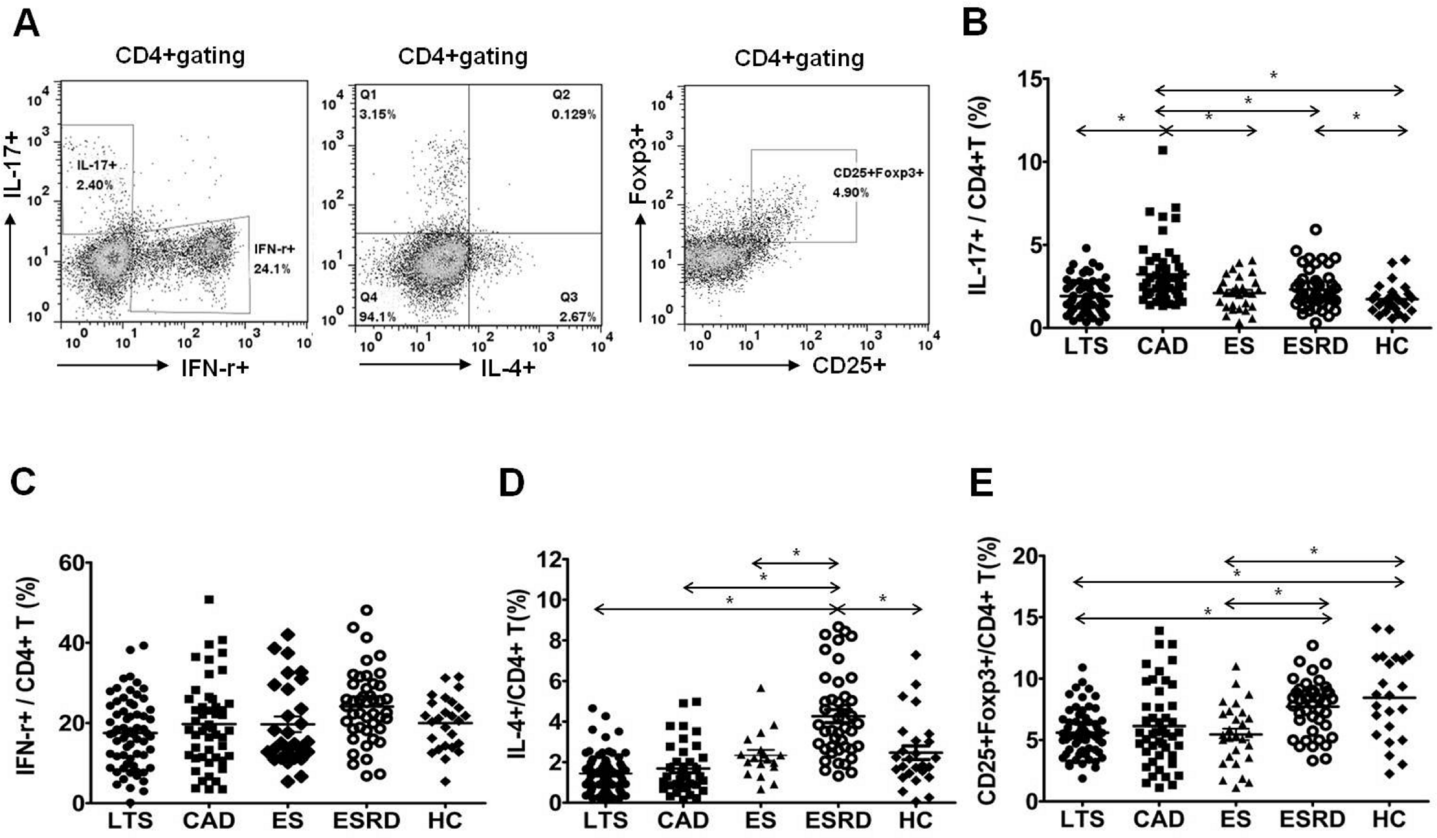
Distribution of Th1, Th2, Th17 and Treg subpopulations out of CD4^+^ T lymphocytes. **(A)** PBMCs were stained with anti-CD4 PE-cy7, anti-CD25 APC, anti-IFN-γ FITC, anti-IL-17 PE, anti-IL-4 APC and anti-Foxp3 FITC. CD4+ T cells were gated for further analysis. **(B)** The proportion (%) of IFN-γ^+^/CD4^+^ T cells **(C)** IL-4^+^/CD4^+^ T cells **(D)** IL-17^+^/CD4^+^ T cells **(E)** CD25^+^FOXP3^+^/CD4^+^T cells in each patient group. * *P*<0.05 for each comparison. LTS, long term stable; CAD, chronic allograft dysfunction; ES, early stable; ESRD, end stage renal disease; HC, healthy control.

As shown in Fig 2, the percentage of lymphocytes and CD4^+^ T cells in the peripheral blood did not show differences in all groups. The proportions of Th1 (CD4^+^ IFN-γ^+^), Th2 (CD4^+^IL-4^+^), Th17 (CD4^+^IL-17^+^), and Treg (CD4^+^FOXP3^+^) out of CD4^+^ T cells are presented in Fig 3. The percentage of Th17 cells out of CD4^+^ T cells was significantly higher in the CAD group than in all other groups (P<0.001 for all, Fig 3B). In contrast, the proportion of Th1 cells did not show an increase in the CAD group compared to the other groups (Fig 3C). The proportion of Th2 cells did not differ between the LTS and CAD groups. But it was significantly higher in the ESRD group than in all other groups (P<0.001 respectively, Fig 3D). The proportion of Treg cells did not differ between the LTS and CAD groups (P = 0.67), meanwhile, all three transplant groups (LTS, CAD, and ES) showed a lower proportion of Treg cells compared to the ESRD group or the HC group (P<0.05 for each comparison, Fig 3E).

### Comparison of naïve/memory T cells and IL-17 producing cells out of effector memory T cells

**Fig 4:**
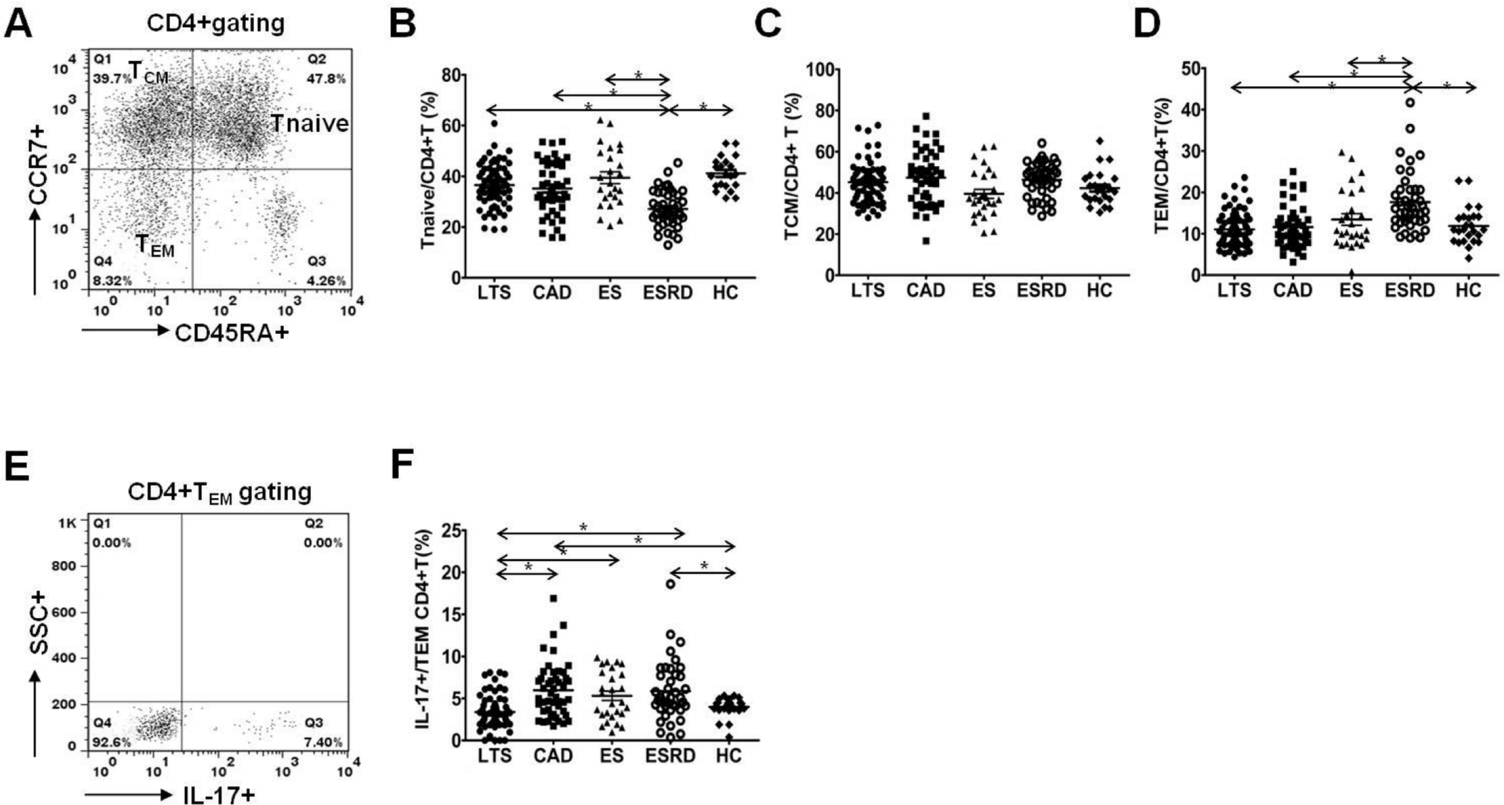
Distribution of T_naïve_, T_CM_, T_EM_ subpopulations of CD4^+^T lymphocytes and IL-17^+^/T_EM_ subpopulations of CD4^+^ T lymphocytes. **(A)** PBMCs were stained with anti-CD4 PE-cy7, anti-CD45RA–FITC, anti-CCR7 APC and anti-IL-17 PE. CD4+ T cells were gated for further analysis. **(B)** The proportion (%) of T_naïve_/CD4^+^ T (CD45RA^*+*^CCR7^+^/CD4^+^ Tcells) **(C)** T_CM_/CD4^+^ T (CD45RA^–^CCR7^+^/CD4^+^Tcells) **(D)** T_EM_/CD4^+^ T (CD45RA^–^CCR7^–^/CD4^+^ Tcells) **(E)** After surface staining with CD45 and CCR7 mAbs, analysis of IL-17 in CD4^+^ T cell subsets by intracellular flow cytometry was done. **(F)** The proportion (%) of IL-17^+^/T_EM._ in each patient group. * *P*<0.05 for each comparison. LTS, long term stable; CAD, chronic allograft dysfunction; ES, early stable; ESRD, end stage renal disease; HC, healthy control.

Staining of peripheral blood T cells with antibodies to CD45RA and CCR7 showed three subsets of CD4^+^ cells; one naïve CD45RA^+^CCR7^+^ subset (T_naive_) and two memory subsets, CD45RA^–^CCR7^+^ central memory T cells (T_CM_) and CD45RA^–^CCR7^–^ effector memory T cells (T_EM_) (Fig 4A). No difference was detected in the percentage of T_naive_ (P = 0.48), T_CM_ (P = 0.37) and T_EM_ (P = 0.64) between the LTS and CAD groups (Fig 4B–4D). However, the percentage of T_naive_ showed a significant decrease in ESRD patients compared to other patient groups (P<0.001 for each comparison, Fig 4B). In contrast, T_EM_ showed a significant increase in the ESRD group compared to other groups (P<0.001 for each comparison). The proportion of IL-17 producing cells was significantly higher in the CD4^+^ T_EM_ subset in patients of the CAD group compared to patients of the LTS group (P<0.001, Fig 4E and 4F).

### Comparison of Th17 associated chemokine receptor expression in CD4^+^ T cells

**Fig 5:**
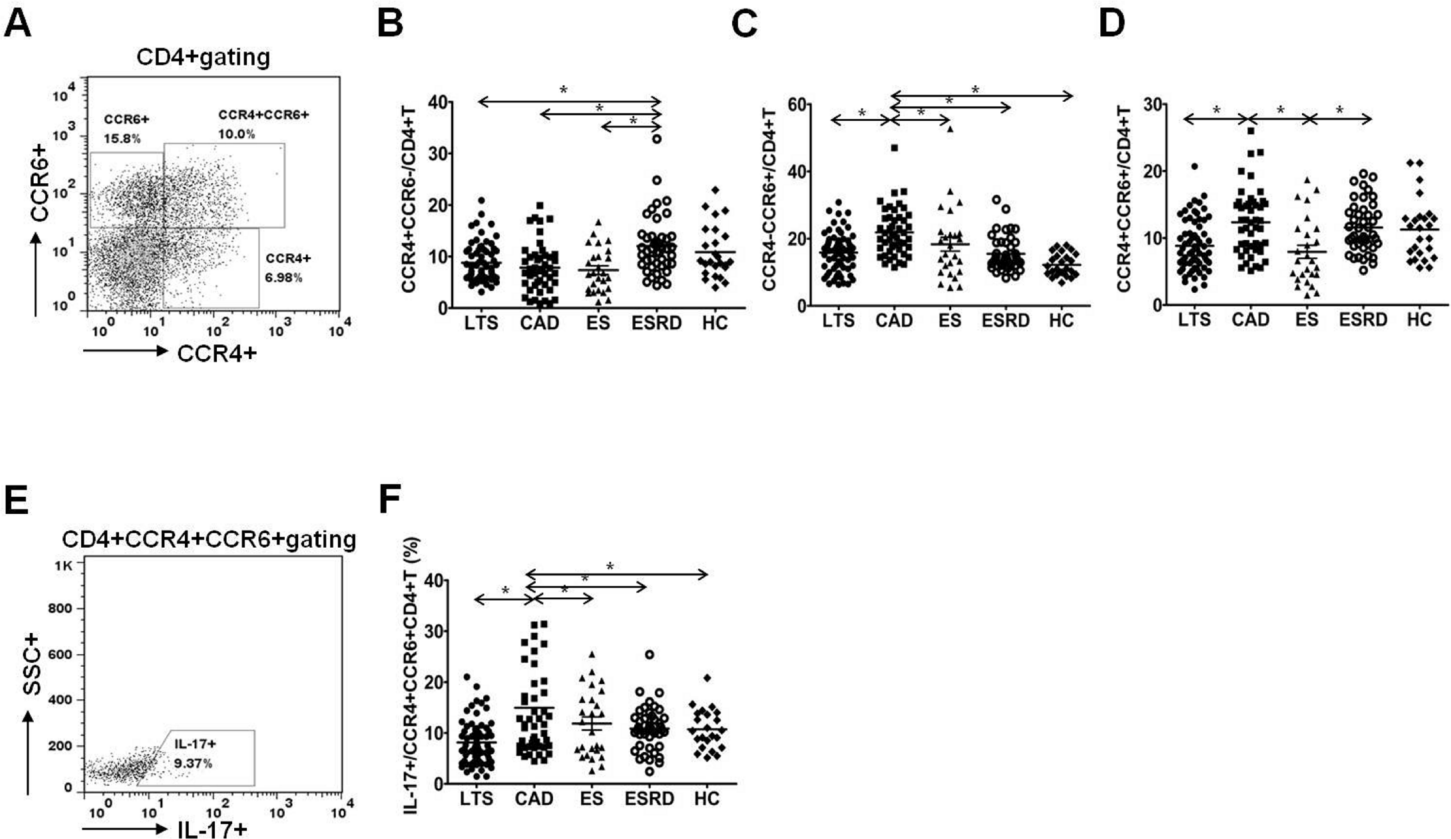
Distribution of chemokine receptor CCR4^+^CCR6^–^, CCR4^–^CCR6^+^ and CCR4^+^CCR6^+^ subpopulations of CD4^+^ T lymphocytes. **(A)** PBMCs were stained with anti-CD4 PE-cy7, anti-CCR4 PE, anti-CCR6 APC and anti-IL-17 FITC. CD4+ T cells were gated for further analysis. **(B)** The proportion (%) of CCR4^+^CCR6^–^/CD4^+^ T cells **(C)** CCR4^–^CCR6^+^/CD4^+^ T cells **(D)** CCR4^+^CCR6^+^/CD4^+^ T cells in each patient group. **(E)** After surface staining with anti-CD4, CCR4 and CCR6 mAbs, analysis of IL-17 in CD4^+^ T cell subsets by intracellular flow cytometry was done. **(F)** The proportion (%) of IL-17^+^/CCR4^+^CCR6^+^CD4^+^ T cells in each patient group. * *P*<0.05 for each comparison. LTS, long term stable; CAD, chronic allograft dysfunction; ES, early stable; ESRD, end stage renal disease; HC, healthy control.

Fig 5A is the representative figure of flowcytometric analysis for CCR4^+^ or CCR6^+^ cells in CD4^+^ T cells. As shown in Fig 5B, the percentage of CCR4^+^CCR6^-^/CD4^+^ T cells did not differ between LTS and CAD group (P = 0.21) and it was increased in the ESRD group compared to the three transplant group (P<0.05 for each comparison). In contrast, the percentage of CCR4^-^CCR6^+^/CD4^+^ T cells showed significant increase in the CAD group compared to the LTS group (P<0.05, Fig 5C). The percentage of CCR4^+^CCR6^+^/CD4^+^ T cells was also higher in the CAD group than in the LTS group or in the ES group (P<0.05 for each comparison, Fig 5D). In addition, the proportion of IL-17 producing cells out of CCR4^+^CCR6^+^CD4^+^ T cells was significantly higher in the CAD group than in the LTS group as well as than in the other groups (P<0.05 for each comparison, Fig 5E).

### Comparison of Th17 associated cytokine level and the expression of markers

**Fig 6:**
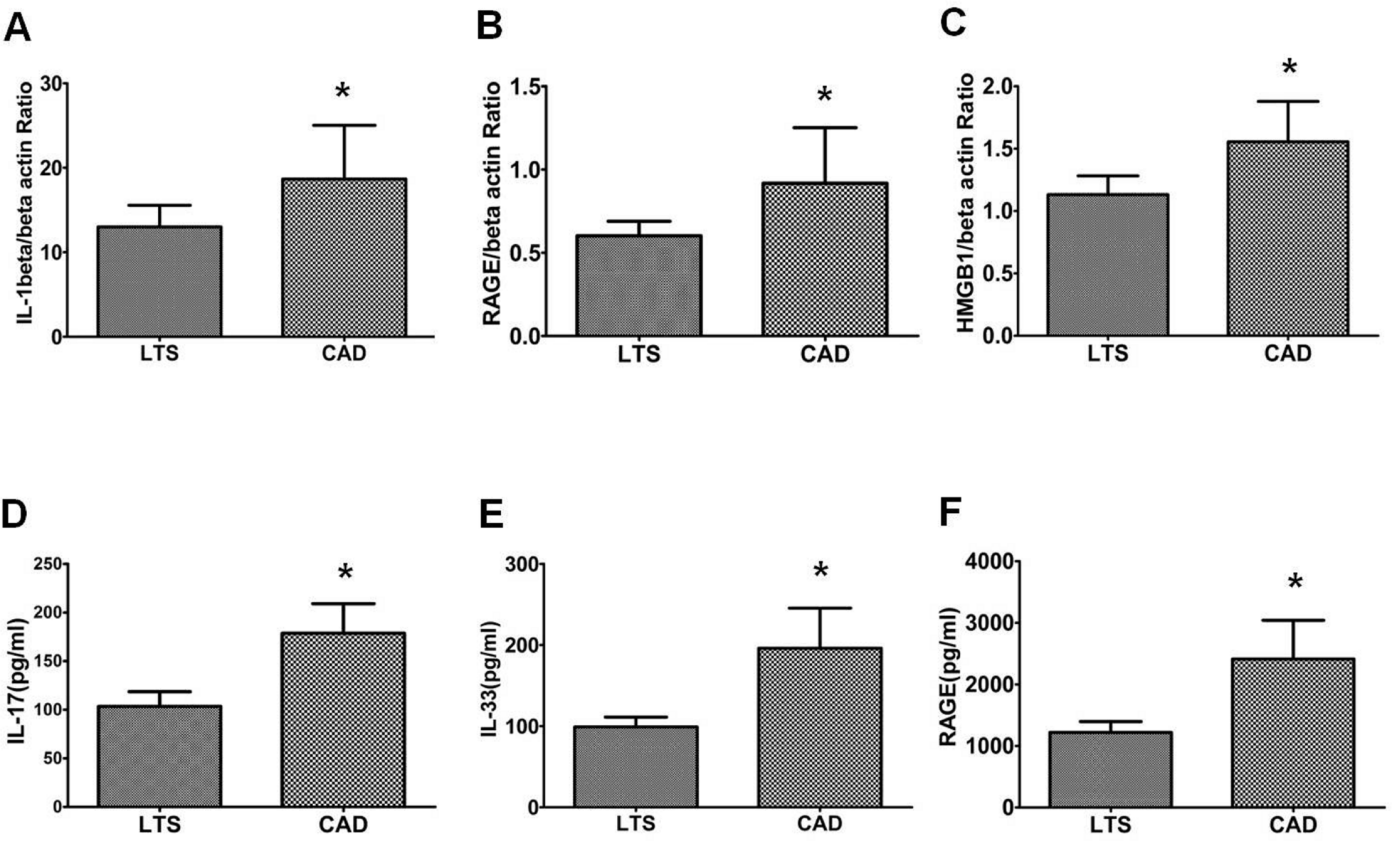
Expression of Th17 pathway molecules and serum Th17 associated cytokine level in LTS and CAD group. The expression of **(A)** *IL-1beta* **(B)** *RAGE* **(C)** *HMGB1* mRNA was measured using real-time PCR. In addition, concentrations of **(D)** IL-17 **(E)** IL-33 and **(F)** RAGE were determined using ELISA in the serum of LTS and CAD patient group. Bars show the means. * *P*<0.05 vs. LTS. CAD, chronic allograft dysfunction; LTS, long term stable.

After PBMC were stimulated with PMA and ionomycin, the expression of mRNA for IL-1beta, RAGE, and HMGB1 was determined using real-time polymerase chain reaction. As shown in Fig 6A–6C, the expression of IL-1beta mRNA, RAGE mRNA and HMGB1 mRNA were suppressed in the LTS group compared to the CAD group (IL-1beta, P = 0.05; RAGE, P = 0.03; HMGB1, P = 0.07, vs. LTS). We also measured Th17 associated cytokine serum level isolated from same peripheral blood used for flowcytometric analysis and the results showed a similar trend (Fig 6D–6F); hence IL-17, IL-33, and RAGE levels showed a significant decrease in the LTS group compared to the CAD group (P<0.05 for all comparison).

### The effect of IL-17 on inflammatory cytokine production and pro-fibrotic gene expression in cultured human renal proximal tubular epithelial cells

**Fig 7:**
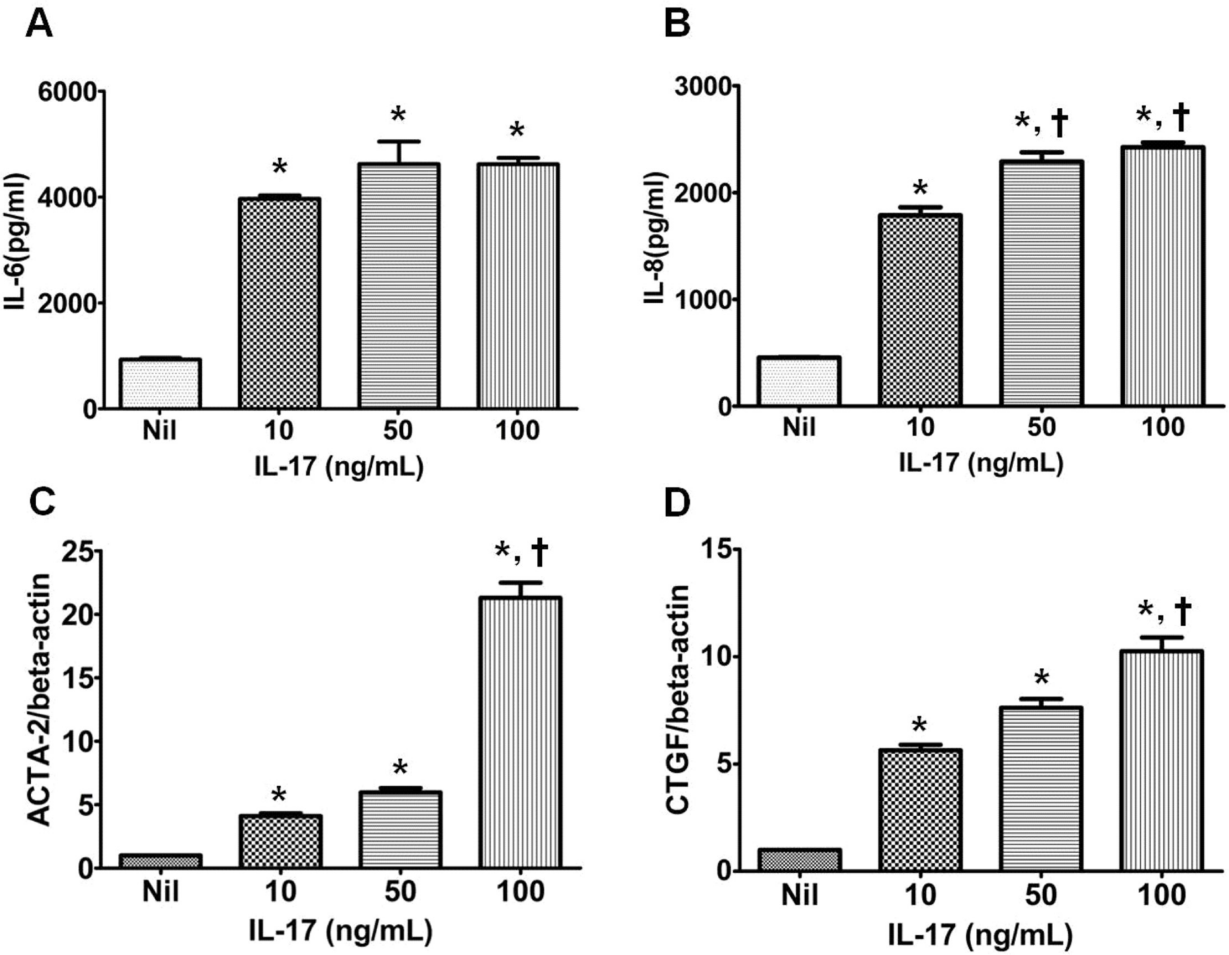
Expression of acute and chronic injury markers in renal tubular cells treated with recombinant human IL-17. Renal tubular epithelial cell were cultured with rhIL-17 (0, 10, 50, or 100 ng/ml) for 72 hours, and the production of **(A)** IL-6 and **(B)** IL-8 was measured using ELISA. Renal tubular epithelial cell were cultured with rhIL-17 (0, 10, 50, or 100 ng/ml) for 72 hours, and the expression of **(C)** *ACTA-2* and **(D)** *CTGF* mRNA, relative to *β-actin*, was measured using real-time polymerase chain reaction. Bars show the means. *P<0.05 vs. Nil, ^†^P<0.05 vs. IL-17 10.

HRPTEpiCs were cultured with three different doses of rhIL-17 (10, 50, 100 ng/ml) and we measured the IL-6 or IL-8 level after 72-hour incubation. Fig 7A and 7B showed that all three doses of rhIL-17 treatment induced significantly increased production of IL-6 or IL-8 in a dose-dependent manner (P<0.05 vs. Nil for each comparison). The effect of IL-17 on pro-fibrotic gene expression in HRPTEpiCs was examined as well. As shown in Fig 7C and 7D, all three doses of rhIL-17 significantly increased the expression of ACTA-2 (α-SMA) and CTGF (connective tissue growth factor) genes in a dose-dependent manner as well (P<0.05 vs. Nil for each comparison).

## Discussion

In this study, we focused on the differences in CD4^+^ T cell phenotype between KTRs with chronic allograft dysfunction and KTRs with long-term stable allograft function. As a result, we identified the up-regulation of the Th17 cell phenotype in KTRs with chronic allograft dysfunction not only in comparison to long-term stable KTRs but also in comparison to ESRD patients and other control groups. This suggests a significant role of the Th17 cell pathway in the progression of chronic allograft dysfunction in kidney transplantation.

The first issue of this study was how to include and classify the patient populations. For the CAD group, we selected KTRs who were at least 2 years post-KT and showed not only morphological evidence of presence of IF/TA but also functional deterioration, usually defined as estimated glomerular filtration rate (eGFR) below 40 mL/min/1.73 m^2^ [23,24]. However, the definition of long-term stable patients is ambiguous and has not been clearly established. In this regard, we tried to select patients who showed the most favorable clinical outcome in our center. We decided to choose patients who were at least 10 years post-transplantation and showed higher MDRD eGFR compared to the average value of eGFR in our center for the long-term stable good outcome group. In addition, we included control groups such as ESRD, early stable KTRs, and healthy control group, which may show differentiated immunologic characteristics compared to the LTS or the CAD group [18,25].

Second, we investigated the T cell phenotype using multi-color FACS in each group and compared the results, and we found that the most prominent finding was the significant increase in the proportion of Th17 cells in the CAD group compared to the LTS group. Previously, we reported that uremic condition induced by renal dysfunction can be associated with the up-regulation of IL-17 producing effector T cells [25]. However, in comparison between the ESRD and CAD groups, the proportion of Th17 cells was significantly increased in the CAD group although renal function was more severely deteriorated in the ESRD group. In addition, the LTS group showed a significantly lower proportion of Th17 cells compared to the ES group in spite of similar allograft function between the two groups. The above findings suggest that the immunologic process rather than renal dysfunction may be mainly involved in the up-regulation of Th17 cells in the CAD group and in the down-regulation of Th17 cells in the LTS group.

In addition, we examined the naïve and memory T cells by staining peripheral blood T cells with antibodies to CD45RA and CCR7. The entire cohort of T_naïve_, T_CM,_ and T_EM_ cells did not show any difference between the CAD and LTS groups. But interestingly, the proportion of IL-17-producing cells among T_EM_ cells was significantly increased in the CAD group. This suggests that the increase in Th17 cells may result from an increase in activated T cells, and not in quiescent T cells. In the analysis of the control groups, the T_naïve_ cell percentage was significantly lower in the ESRD group; in contrast, the T_EM_ cell percentage was significantly increased in the ESRD group compared to all other groups. T_naïve_ cells usually represent the immune cell pool that could be recruited in active infectious conditions; in contrast, T_EM_ cells represent actively differentiated immune cells [26,27]. Hence, these results suggest diminished immune cell pool and non-specific activation of T cells in ESRD patients and they are fully consistent with the findings in our previous report [25].

Interestingly, CD4^+^CD25^high^FOXP3^+^ T cells, so called Treg cells did not show any difference in the CAD group compared to other groups, which was contradictory to the previous reports about its significant role in kidney transplantation [5,28,29,30]. There could be various reasons for this. First, most patients in both groups took a calcineurin inhibitor such as Tac or CsA, which can decrease the proportion of this T cell type [31,32,33]. Indeed, all three transplant recipient groups (LTS, CAD, and ES) showed a significantly lower proportion of Treg cells out of CD4+ T cells compared to the ESRD or the HC group, in which the patients did not take any immunosuppressants. Second, chronic vascular inflammatory status in patients of the LTS group induced by old age, underlying renal disease, and long-term immunosuppressed state, may have affected the down-regulation of Treg cells in these patients [34,35,36].

Third, we investigated the expression of chemokine receptor on T cells. It is well known that transplant allograft can affect the expression of various chemokine receptors, and CCR4 or CCR6 expression regulates the migration of inflammatory and regulatory T cells [37,38]. Especially, IL-17 producing CD4+ T cells can express both CCR4 and CCR6 [39]. As expected, the percentage of T cells expressing both CCR4 and CCR6 was increased in the CAD group compared to the LTS group and IL-17 production by these cells was increased as well. In contrast, the frequency of CCR4^+^CCR6^–^/CD4^+^T cells did not differ significantly between the CAD and LTS groups, and interestingly, these cells were significantly decreased in the three transplant groups (LTS, CAD, and ES) compared to the non-transplant groups (ESRD and HC). In a previous report, it was demonstrated that in vitro derived Th2 lymphocyte lines selectively express CCR4, CCR8, and CCR3 [40,41]. Moreover, our previous in vivo and in vitro studies showed a significant decrease in the Th2 cell percentage after the initiation of an immunosuppressant [18]. Hence, it is possible that the use of an immunosuppressant in three transplant groups may have resulted in the decrease in CCR4^+^CCR6^–^/CD4^+^T cells.

Next, we compared the expression of markers associated with the development of Th17 cells between the CAD and LTS groups. IL-1beta plays a significant role in the development of Th17 cells [42], while HMGB1 is a potent inducer of pro-inflammatory cytokines, such as IL-1beta and IL-6, which are considered to be crucial mediators in the induction of Th17 cells [43]. Our study showed that HMGB1 and IL-1beta expression was up-regulated in the CAD group compared to the LTS group. On comparison of production of Th17 associated cytokines, IL-17, IL-33, and RAGE levels were significantly higher in the CAD group compared to the LTS group. All of the above findings are consistent with the increased proportion of Th17 cells observed in the CAD group in FACS analysis.

Finally, we tested the effect of representative Th17 cytokine, IL-17 on the renal tubular epithelial cells in vitro to prove direct damage to renal tubular epithelial cells caused by the augmented activation of Th17 pathway. We selected IL-6 and IL-8 secretion by renal tubular epithelial cells as acute injury markers and used the expression of pro-fibrotic markers such as CTGF or ACTA-2 as chronic damage markers in renal tubular epithelial cells [44,45]. As a result, we found a dose-dependent increase in both acute and chronic damage markers in HRPTEpiCs induced by IL-17. This result is fully consistent with that in the previous reports which suggest chronic renal tubular injury induced by Th17 associated cytokines, and may partially explain the development of chronic allograft dysfunction by IL-17 [46,47].

It is possible that acute process by the activation of Th17 may precede the chronic allograft dysfunction. In our previous report, infiltration of Th17 was significant in more severe allograft rejection. (Chung et al, Immunology 2012;136:344, Chung et al, Exp Mol Med 2011) However, in our preliminary results using peripheral blood, the proportion of Th17 did not show increase in patients with acute rejection compared to patients with normal biopsy. (S1 Fig). Above findings suggest that even though activation of Th17 may initiate in allograft tissue, the increase may not evident at this state, and only after it progress to chronic change, which may resulted in chronic dysfunction, increase of Th17 cell became evident in peripheral blood.

This study may have some limitations. For example, we did not perform allograft biopsy in the LTS group; hence it is possible that chronic damage may exist in allograft tissue from patients in this group. However, considering the contrasting clinical status between the LTS and CAD groups, it did not indicate some overlap between the LTS and CAD groups, and chronic change in the allograft tissue, if any, may be far less severe in the LTS group compared to the CAD group. Second, we did not show the impact of the Th17 pathway on the future clinical outcome. Sequential monitoring in the prospective cohort may be required to clarify this issue.

In conclusion, KTRs with chronic allograft dysfunction showed a significantly different T cell phenotype compared with KTRs with a long-term stable outcome and ES or ESRD controls. The most important finding was a significant up-regulation of the Th17 cell phenotype in PBMCs from KTRs with chronic allograft dysfunction. In addition, Th17 associated pro-inflammatory cytokine, IL-17 induced acute and chronic injury markers in HPRTEpiC in a dose dependent manner, which suggests its role in the development of allograft injury. The results of this study might indicate that there is a need to develop diagnostic and therapeutic tools targeting the Th17 pathway for detection and also prevention of the development of chronic allograft dysfunction in KTRs.

## Supporting Information

S1 Fig Distribution of IL-17 subpopulations out of CD4^+^ T lymphocytes in normal biopsy and T cell mediated rejection (TCMR) patient group. (PDF)

S2 Fig Raw data of target cells in each patient group. PBMC from each patient group were stimulated for 4h ex vivo with PMA and ionomycin in the presence of Golgi Stop. The percentage of target cells was measured by flowcytometry.
(PDF)
