# Supplementary figures and images for: Increase of Th17 Cell Phenotype in Kidney Transplant Recipients with Chronic Allograft Dysfunction
Source: PLoS One. 2015 Dec 30;10(12):e0145258. doi: 10.1371/journal.pone.0145258 (PMC4696852; doi:10.1371/journal.pone.0145258)

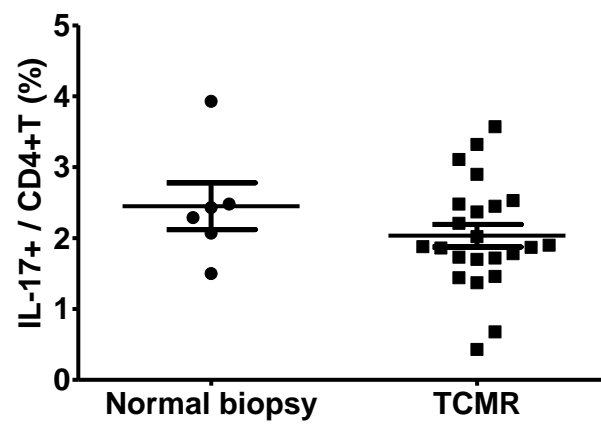

TCMR, T cell mediated rejection

Supplement: S1 Fig — (PDF) [file pone.0145258.s001.pdf]
