# Supplementary material for: Increase of Th17 Cell Phenotype in Kidney Transplant Recipients with Chronic Allograft Dysfunction
Source: PLoS One. 2015 Dec 30;10(12):e0145258. doi: 10.1371/journal.pone.0145258 (PMC4696852; doi:10.1371/journal.pone.0145258)
Supplement: S2 Fig — PBMC from each patient group were stimulated for 4h ex vivo with PMA and ionomycin in the presence of Golgi Stop. The percentage of target cells was measured by flowcytometry. (PDF) [file pone.0145258.s002.pdf]

Group	IL-17+/CD4+(Th17)	IFN-r+/CD4+(Th1)	IL-4+/CD4+(Th2)	CD25+Foxp3+/CD4+ T (Treg)	CCR4+ CCR6+/CD4+ T	IL-17+ / CCR6+ CCR4+CD4+T	Effector memory CD4+ T	IL-17+/Effector Memory CD4+ T
LTS	1.13	14.7	0.582	2.89	6.97	11.9	6.58	1.94
LTS	2.37	18.8	0.901	4.23	7.69	9.48	11.4	2.38
LTS	1.73	7.64	1.5	8.73	5.57	4.28	9.72	4.97
LTS	2.91	21.6	2.54	4.21	15.6	7.97	9.73	7.79
LTS	1.63	17.4	1.76	4.14	14.9	4.48	8.69	1.83
LTS	2.81	18	1.07	6.49	9.39	8.94	11.5	4.83
LTS	1.7	18.8	1.97	5	6.38	4.03	7.39	2.85
LTS	1.82	9.33	2.45	3.06	7.07	2.9	7.33	1.86
LTS	0.857	12.3	0.609	3.84	8.48	6.77	7.25	3.88
LTS	1.85	22.1	1.65	5.79	10.6	5.77	15	2.75
LTS	1.58	6.82	1.55	3.23	10.6	1.47	6.82	5.37
LTS	2.6	18.4	1.32	5.62	3.46	19.1	7.45	4.17
LTS	1.76	20.2	1.73	2.75	8.77	11.8	14.3	4.73
LTS	1.81	28.6	2.24	7.43	4.65	9.38	11.7	0
LTS	3.38	11.7	2.35	5.03	12.7	3.34	9.29	6.28
LTS	2.82	4.66	0.889	6.3	6.7	7.65	8.05	3.39
LTS	0.422	17.5	0.861	5.81	20.7	3.81	14.6	2.25
LTS	1.46	17.2	3.23	9.71	6.36	15.9	8.53	3.97
LTS	3.04	39.3	1.42	5.69	13.7	5.69	18	2.62
LTS	1.98	26.3	4.26	1.87	12.4	6.12	20.7	6.08
LTS	0.788	5.43	0.119	5.08	5.41	7.61	8.62	2.39
LTS	2.38	16	1.7	3.4	7.75	5.03	9.99	3.28
LTS	1.9	25	0.437	5.26	7.8	11	12.6	6.05
LTS	2.3	5.97	1.76	6.69	11.7	9.33	6.14	1.09
LTS	2.73	23.3	4.65	4.01	12.7	9.64	13.2	6.28
LTS	0.355	22.9	0.7	3.54	2.33	2.33	23.6	2.57
LTS	1.55	8.69	0.796	10.9	14.4	14.4	8.25	3.32
LTS	0.585	3.81	0.2	5.592	14.1	6.08	16.2	3.62
LTS	1.51	31.6	1.33	6.58	10	6.58	7.77	3.371
LTS	0.721	13.4	0.5	3.36	6.69	5.59	9.13	1.63
LTS	0.622	12	0.387	4.05	4.98	8.77	8.16	2.09
LTS	1.91	15.4	1.35	8.49	5.11	9.31	9.92	4.38
LTS	1.08	28.7	1.6	3.1	5.38	10.3	16.1	1.93
LTS	2.47	12.3	1.81	5.27	5.04	12.2	10.4	8.07
LTS	2.71	17.3	2.14	5.6	10.7	1.47	9.37	4.94
LTS	1.2	7.73	0.864	8.57	6.63	6.43	13.2	0
LTS	3.69	20.9	1.21	3.55	6.24	6.54	16.5	1.61
LTS	1.42	14	0.591	6.46	4.82	6.16	10.8	2.89
LTS	3.84	27.9	0.646	6.43	2.96	16.8	12.8	2.89
LTS	1.05	17.5	0.89	7.06	3.96	10.7	7.24	4.16

LTS	0.651	27.1	0.262	4.98	6.23	7.33	13.1	2.95
LTS	1.59	38.2	3.6	7.4	8.38	10.3	21.5	0
LTS	1.09	11.1	0.428	5.15	9.58	2.65	18.4	3.371
LTS	0.967	18.5	0.923	5.53	13.2	10.9	10.6	2.16
LTS	1.86	7.58	1.15	7.73	16.3	3.96	6.94	2.64
LTS	3.45	30.1	1.22	3.83	7.95	3.16	14	7.29
LTS	2.63	16.2	2.46	9.18	15.3	4.43	5.64	2.11
LTS	1.56	8.77	1.36	4.71	6.62	13.9	13.5	0.973
LTS	2.37	2.99	0.562	3.8	13.6	9.27	11.9	3.36
LTS	3.92	15.5	2.1	5.3	7.7	6.5	5.3	3.11
LTS	1.5	17.2	0.246	5.592	7.37	5.42	7.65	5.88
LTS	4.8	24.3	2.47	5.04	9.94	3.81	16.9	7.89
LTS	2.43	22.1	3.51	9.28	13	11.5	8.93	3
LTS	2.51	16.6	1.01	7.7	7.1	6.11	5.83	3.59
LTS	1.94	21.7	1.99	5.58	6.68	3.76	9.57	0
LTS	2.55	9.74	2.46	4.79	14	6.79	5.73	8.08
LTS	0.893	24.5	2.53	6.8	4.14	14.9	15.6	2.82
LTS	2.69	31.1	1.44	5.19	12.8	7.11	11.4	3.22
LTS	1.15	17.7	0.53	6.04	6.83	4.31	13.5	1.82
LTS	0.939	25.4	0.354	4.32	9.52	6.82	14.4	2.17
LTS	3.32	0.115	0.23	6.23	8.74	3.91	4.38	3.36
LTS	3.49	12	0.985	5.99	6.21	7.51	8.31	3.93
LTS	0.859	21	0.343	6.09	5.53	15.4	12.5	2.49
LTS	1.39	8.82	1.87	5.44	9.13	16.3	5.21	2.33
LTS	1.74	7.84	1.54	5.58	4.13	21	5.33	3.382
LTS	0.372	26.1	0.413	7.58	9.94	9.94	19.1	0.504
LTS	1.35	28.2	1.91	5.02	10.4	11.3	11.2	2.55
CAD	10.7	18.7	0.321	1.51	5.12	31.4	22.4	7.4
CAD	1.72	3.75	1.09	4.55	19.3	8.6	11.6	11
CAD	1.33	19.1	2.02	2	8.78	8.7	11.8	3.11
CAD	3.57	10.6	0.863	4.72	8	7.4	12.2	5.61
CAD	1.7	23.1	0.492	12.8	14.6	5.89	9.16	2.32
CAD	3.6	16.4	1.58	2.56	5.57	27.5	9.19	8.24
CAD	2.03	24	0.374	13.9	11.4	8.01	12.8	3
CAD	3.225	19.72	2.22	6.13	12.3	14.9	11.61	5.969
CAD	1.38	12	1.64	4.06	12.73	5.7	15.9	5
CAD	1.44	11.8	0.695	9.53	22.8	5.72	9.74	2.2
CAD	2.48	11.4	1.67	5.69	12.4	14.96	11.63	5.95
CAD	4.54	18	1.04	5.62	16.9	6.2	6.83	8.9
CAD	3.96	5.54	3.78	5.07	15.9	8.43	7.9	4.34
CAD	1.38	3.48	0.949	6.13	15.4	11.7	13.7	4.55
CAD	2.66	40.7	1.691	2.03	14.5	11	10.1	1.73
CAD	2.4	24	1.27	6.142	20	5.38	18.8	4.86

CAD	2.03	13.9	1.31	6.136	22.6	4.46	21.6	3.5
CAD	2.06	39.6	1.16	6.45	9.1	24.5	11.6	5.988
CAD	3.01	26	1.802	3.76	14.9	7.14	9.46	8.12
CAD	3.01	26	1.691	3.76	14.9	7.14	9.46	8.12
CAD	1.57	8.55		2.8	12.15	14.8	11.64	5.969
CAD	2.91	35.8	4.98	3.08	1	15.06	15	2.29
CAD	2.51	22.1	2.01	3.23	11.5	12.8	12.8	7.07
CAD	2.05	33.2		2.1	9.2	16.8	16.7	7.06
CAD	2.38	3.48	1.69	6.136	15.4	11.7	13.7	4.55
CAD	3.48	50.8	4.9	11.2	14.13	13.3	8.08	2.01
CAD	2.17	20.5	0.599	4.89	16.9	8.22	10.7	2.27
CAD	2.16	24.8	2.77	1.34	15.11	12.7	7.79	7.3
CAD	3.16	10.1	0.927	8.97	12.7	9.22	8.36	6.77
CAD	4.15	5.44	0.15	5.65	12.7	23.6	3.17	16.9
CAD	4.25	23.2	1.3	5.29	10.7	7.2	9.62	3.5
CAD	3.02	13.3	1.74	11.5	9.29	17.2	6.58	6.2
CAD	2.61	22	0.828	9.78	26	11.3	12	2.83
CAD	1.894	11.5	3.16	1.13	12.6	29	4.82	4.67
CAD	5.87	7.99		4.4	11.6	16.1	6.98	3.65
CAD	6.7	18.1	3.52	7.64	5.75	14.2	6.42	8.21
CAD	6.62	6.67	0.893	6.58	14.8	12.8	4.57	6.17
CAD	1.58	14.3	0.726	5.6	15.5	4.62	6.86	2.27
CAD	2.74	16.4	3.78	5.48	12.3	7.32	8.42	2.7
CAD	4.72	17.1	1.43	5.54	6.44	7.43	9.79	7.06
CAD	2.27	28.2		4.54	8.14	9.92	7.88	4.52
CAD	2.01	32.2		6.81	9.2	7.51	5.35	6.9
CAD	3.225	19.72	0.726	6.136	12.12	14.93	11.62	5.969
CAD	3.23	23.2	2.5	10.6	7.3	48.9	11.5	13.7
CAD	2.71	19.6		4.72	8.3	47.9	13.7	10.7
CAD	2.96	11.2	0.224	9.94	9	19.7	19.5	7.34
CAD	7	17	2.8	7.78	15.1	14.2	20.9	8.87
CAD	1.83	18.3	1.72	6.81	8.25	20.2	10.4	4.1
CAD	3.67	22.2	1.67	7.71	6.19	17.9	11.9	5.16
CAD	4.03	36.5	1.68	9.85	7.08	27.8	13.4	4.62
CAD	7.25	26.5	1.7	12.8	11.2	31.2	21.8	12.6
CAD	2.73	37.5		6.48	5.57	26.1	25	6.54
ES	2.73	23.5	2.38	11	4.55	22	0.866	7.84
ES	2	28.4	3.45	7.35	2.75	18.3	9.43	1.89
ES	1.1	15.3	3.12	1.86	7.36	6.72	15.8	4.38
ES	2.42	13.4	2.334	1.12	15.7	4.85	10.8	3.31
ES	1.04	13.4	2	3.07	5.33	7.54	9.57	3.66
ES	1.5	42	2.34	9.6	9.45	6.63	24.8	3.91
ES	2.39	32.7	1.97	3.56	6.61	25.5	28.2	8.09

ES	2.15	38.6	2.3	5.08	16.2	13.8	14.6	2.82
ES	4.07	10.9	5.66	7.99	13	19.5	7.73	4.56
ES	0.72	6.65	2.333	4.19	11.4	7.18	9.91	2.12
ES	3.37	11.2	2.6	6.67	5.6	5.6	13.4	5.61
ES	2.43	29.5	1.91	6.77	18.7	16.6	13.44	5.308
ES	3.56	19.8	1.77	8.7	8.94	7.23	10.8	3.2
ES	3.93	13.5	2.334	5.85	4.77	13.8	20.5	9.12
ES	3.28	11	1.41	6.31	7.94	11.85	14.4	9.12
ES	2.76	12.8	0.664	8.13	8.02	5.24	6.8	6.19
ES	2.11	12.8	2.13	5.41	6.63	14.1	8.77	6.19
ES	3.11	31	2.37	4.57	7.946	11.85	12.4	8.68
ES	2.22	17.6	0.909	5.01	10.5	5.21	8	9.35
ES	1.14	5.38	2.6	5.37	3.41	3.41	10.4	3.45
ES	1.3	32.5	2.068	1.71	17.2	10.2	23.2	3.07
ES	1.59	21.7	1.28	1.54	11.4	6.92	20.9	0.97
ES	2.07	14.8	2.368	3.65	1.79	16.5	7.23	7.34
ES	1.37	15	3.83	7.24	1.46	20.3	9.91	6.04
ES	2.29	18.2		7.12	4.57	20.8	7.57	9.36
ES	1.26	10.1	2.21	5.17	2.73	15.4	19.9	9.86
ES	0.575	37.4	2.335	5.52	3.83	12.2	29.7	1.64
ES	0.346	11.2		3.09	4.63	2.58	7.25	1.54
ESRD	1.05	26	3.84	9.03	9.2	9.73	15	5.867
ESRD	1.96	21.4	8.44	8	12.5	10.9	25.5	5.86
ESRD	1.69	41.3	4.6	3.32	10.5	11.9	35.4	0.35
ESRD	1.78	16	2.05	6.62	7.32	11.4	13.3	0.76
ESRD	1.46	6.79	2.59	7.18	9.69	4.16	15.1	0.91
ESRD	1.18	26.8	3.52	8.35	17.9	6.43	27.7	3.62
ESRD	1.74	25.8		8.48	19.1	12.9	14.7	5.866
ESRD	3.01	19.9	3.08	7.48	13.5	9.82	17.63	7.71
ESRD	0.72	12.2	4.18	9.15	6.79	9.81	17.25	4.26
ESRD	1.17	25.9	3.45	7.717	7.02	10.4	18.77	3.77
ESRD	2.39	25.1	7.1	8.98	10	5.13	12.7	11.7
ESRD	2.71	25.5	1.89	9.59	9.95	7.35	13.7	7
ESRD	2.05	22.2	6.16	6.66	13.5	12.8	13.5	8.51
ESRD	1.65	20.4	4.98	10	11.7	6.07	13	4.47
ESRD	1.9	18.5	1.94	12.7	19.6	10.1	14.9	5.71
ESRD	2.84	29.3	2.45	4.48	13.4	10.6	10.8	8.65
ESRD	5.92	26.6	6.16	6.21	10.2	12.6	19.3	3.59
ESRD	3.92	7.25	1.6	8.84	11.56	10.85	17.7	12.6
ESRD	1.46	14.3	4.28	6.91	9.06	7.02	17.94	6.14
ESRD	2.94	31.8	5.03	7.39	16.2	10.5	11.5	10.6
ESRD	4.64	31.5	3.56	8.44	17.2	11.2	25.1	18.6
ESRD	4.19	32.4	4.95	8.07	16.1	16.1	20.7	5.872

ESRD	1.85	28.6	5.08	4.93	14.2	9.24	29.7	5.865
ESRD	0.3	36.8	8.29	9.31	6.96	14.4	20.7	2.19
ESRD	1.26	21.4	2.77	10.7	11.5	11.1	16.1	4.11
ESRD	1.04	9.7	1.49	9.85	13.8	5.32	9.26	7.81
ESRD	3.98	22.3	3.9	11.2	8.82	12.3	18	8.62
ESRD	1.72	22.4	4.61	4.99	5.18	13	12	4.25
ESRD	1.02	16	2.91	4.99	6.17	2.42	13.2	7.62
ESRD	1.98	18.9	5.94	5.2	12.4	10.2	29	4.08
ESRD	2.08	21.1	3.54	4.56	7.09	15.2	9.76	2.93
ESRD	3.9	24.1	1.32	8.85	18.5	10.85	16.8	5.36
ESRD	0.85	10.9	7.55	8.95	9.97	7.54	9.93	4.75
ESRD	4.17	32.1	3.69	7.717	12.7	13.3	16.3	6.11
ESRD	2.59	25.6	2.76	3.47	9.73	10.4	15.2	5.1
ESRD	2.46	18.1	5.23	5.73	11.6	4.86	17.63	5.09
ESRD	2.69	43.8	2.64	7.78	10.3	10.6	41.7	5.09
ESRD	4.22	35.6	3.19	8.35	8.58	15	22.7	5.866
ESRD	1.74	25.8	8.21	7.22	9.68	18.1	13	2.36
ESRD	1.36	18.6	2.18	8.29	9.11	14	15.44	1.74
ESRD	1.9	29.8	8.66	8.89	8.55	10	9.01	9.58
ESRD	2.05	29.7	2.79	11.4	15	17.9	16.5	4.09
ESRD	3.53	15.2	4.26	8.36	7.39	4.71	9.09	4.53
ESRD	2.61	22.6	2.64	8.47	14.6	14.6	20.6	5.78
ESRD	2.58	48.1	8.02	4.48	16.2	25.4	20.6	8.65
HC	1.07	13.4	1.45	7.52	7.98	12.6	7.96	4.94
HC	1.88	20.5	2.2	9.35	7.03	14	13.7	3.63
HC	1.97	12.8	1.09	11.9	12.9	8.82	8.27	4.92
HC	1.16	13.9	3.51	8.63	6.75	9.08	6.66	4.41
HC	4.09	14.3	1.55	8.69	18.7	12.5	16.5	3.57
HC	1.81	21.8	2.43	5.41	13.2	10.4	8.17	4.4
HC	1.74	31.2	7.28	11.7	8.46	13.8	11	4.47
HC	3	12.5	1.25	11.8	12.6	10.5	9.63	4.5
HC	0.98	27	1.79	7.33	5.59	7.12	14.1	5.1
HC	1.67	5.41	0.55	7.01	13.6	13.6	12	5.32
HC	3.93	23.8	0.26	9.61	21.2	8.52	22.8	3.8
HC	0.56	25.21	3.39	8.439	5.57	10.69	16.5	4.41
HC	0.83	24.98	3	8.459	8.61	10.71	8.17	3.57
HC	0.6	14.9	2.54	2.25	9.83	5.5	11	4.4
HC	0.74	18.9	1.24	11.5	11.8	5.15	9.63	4.1
HC	1.4	28.93	4.98	14	21.21	7.11	14.1	3.8
HC	1.28	17.2	1.76	4.98	11.27	10.72	12	4.5
HC	1.42	22.6	0.058	4.79	12.9	6.3	22.8	5.32
HC	1.04	31.5	1.84	3.01	7.76	20.8	11.88	4.8
HC	1.38	20.3	5.24	3.73	11.5	9.39	4.09	3.8

HC	1.6	21.8	2.16	8.429	12.9	5.88	7.84	3.983
HC	2.04	16.2	1.64	14.1	9.94	15.6	11	0.38
HC	1.81	11	3.05	6.1	6.48	14.4	13.7	1.88
HC	2.44	21.1	2.24	7.78	6.6	15.1	10.6	3.76
HC	2.16	26.3	5.83	11.2	11.9	9.1	14.2	1.87
HC	2.52	21.7	1.73	11.7	16.7	11	10.6	3.93
